# Nicotine Dependence from Different E-Cigarette Devices and Combustible Cigarettes among US Adolescent and Young Adult Users

**DOI:** 10.3390/ijerph19105846

**Published:** 2022-05-11

**Authors:** Crystal Lin, Shivani Mathur Gaiha, Bonnie Halpern-Felsher

**Affiliations:** 1 Stanford School of Medicine, Stanford University, Palo Alto, CA 94304, USA; clin4@stanford.edu; 2 Reach Lab, Division of Adolescent Medicine, Department of Pediatrics, Stanford University, Palo Alto, CA 94304, USA; gaiha@stanford.edu

**Keywords:** nicotine dependence, e-cigarettes, vaping, adolescents and young adults

## Abstract

E-cigarettes, the most popular tobacco product among adolescents, vary widely in design and nicotine composition; thus, different devices may have different addictive potential. However, few studies examine levels of nicotine dependence across devices among adolescent and young adult (AYA) e-cigarette users. To assess the extent of nicotine dependence among US AYA (ages 13–24) by e-cigarette device type, we conducted a large, national, cross-sectional survey (*n* = 4351) and used the Hooked on Nicotine Checklist (HONC) to assess levels of nicotine dependence among those who had used disposable, pod-based, and/or mods/other e-cigarette devices in the past 30 days. We also examined HONC scores among those who had used combustible cigarettes in the past 30 days, whether with or without using e-cigarettes. Patterns of nicotine dependence were comparable across those who had used a combustible cigarette and/or e-cigarette in the past 30 days, with 91.4% of combustible cigarette users, 80.7% of disposable e-cigarette users, 83.1% of pod-based e-cigarette users, and 82.5% of mods/other e-cigarette users showing signs of nicotine dependence, as measured by endorsing at least one HONC symptom. This pattern persisted when analyses were restricted to e-cigarette only users, with more than 70% of all e-cigarette only past-30-day users endorsing at least one HONC symptom, across all types of devices. A thorough understanding of the extent and presentation of nicotine dependence among AYA will help researchers, public health officials, and clinicians recognize and manage AYA nicotine dependence.

## 1. Introduction

E-cigarettes are the most commonly used tobacco product among adolescents [1]. Most e-cigarettes contain nicotine, a highly addictive substance that can harm the developing brain, making adolescents and young adults (AYA) particularly susceptible to nicotine dependence [2,3]. Due to heterogeneity in design and nicotine composition, e-cigarette devices deliver different levels of nicotine, potentially varying their addictive potential. Types of e-cigarette devices include mods, pods, and disposables, with newer generation e-cigarettes utilizing salt-based formulas that allow for more efficient delivery of nicotine [4]. Further, use of multiple tobacco products has been associated with increased levels of nicotine dependence [5], and multiple e-cigarette device use is common among AYA [6]. While patterns of AYA e-cigarette use have been well characterized [1,7,8,9], few studies address the addictive potential of different e-cigarette devices and levels of nicotine dependence among users, likely due to the novelty of e-cigarettes as a class of tobacco products [10] and ongoing development of measurement tools [11].

The Hooked on Nicotine Checklist (HONC) [12] is an instrument designed to determine the strength and onset of tobacco dependence, and has recently been biochemically validated for e-cigarettes [13]. While originally designed for adolescents, the HONC has since been evaluated for use in adults as well [14]. Unlike other instruments such as the Fagerström Test of Nicotine Dependence [15] which focuses on measuring nicotine consumption, the HONC assesses symptoms such as cravings, concentration, and irritability. Measuring symptoms is advantageous in studying the AYA population, as AYA have been found to develop signs of nicotine dependence prior to the onset of frequent tobacco use [12]. Previous studies utilizing the HONC have found evidence for e-cigarette specific nicotine dependence symptoms [16,17] and that symptoms may vary based on pod-based vs. other e-cigarette device types [18].

We expand on the literature by conducting one of the first national, large-scale, cross-sectional studies examining e-cigarette nicotine dependence across device types among AYA. The aims of this study are: (1) to provide comprehensive evidence on the extent of nicotine dependence among AYAs by e-cigarette device type, with combustible cigarettes as a comparison; (2) to describe nicotine dependence among AYA e-cigarette only users, to ascertain if dependence can arise from exclusive e-cigarette use, without previous nicotine exposure from combustible cigarettes; and (3) to determine if multiple tobacco product use is associated with nicotine dependence. An understanding of the extent of nicotine dependence among AYA is critical for the recognition and management of AYA nicotine dependence in the clinical setting.

## 2. Materials and Methods

### 2.1. Participants

A national cross-sectional survey of 4351 US AYA (ages 13–24) was administered from 6 to 14 May 2020 via Qualtrics. Quota sampling was used to achieve a 1:1:1 ratio of participants aged 13–17, 18–20, and 21–24 years, as well as a 50:50 ratio of ever e-cigarette users to never-users, using the screening question, “Have you ever used an e-cigarette or vape before today?” Using US Census data, the sample was further balanced on sex and race/ethnicity. Participants who completed the survey in less than one-third of the average response time were dropped from the final sample. This study was approved by the Institutional Review Board at Stanford University and follows the American Association for Public Opinion Research reporting guidelines for survey studies. Detailed descriptions of the survey have been previously published [19,20].

### 2.2. Measures

Participants were asked to indicate “yes” or “no” if they had ever used any of the following tobacco products in their entire life: (1) “Cigarettes, even 1 or 2 puffs,” (2) “Disposable pod-based vape like Puffbar or FOGG, even 1 or 2 puffs,” (3) “Pod-based vape like Juul or Suorin, even 1 or 2 puffs,” or (4) “Any other vape like mods, even 1 or 2 puffs.” Participants were asked to indicate past-30-day use of each e-cigarette product or combustible cigarette, in the categories listed above, by indicating the number of days used from 0 to 30. Binary past-30-day use variables were created, with 0 days indicating no past-30-day use. Lifetime use of each product was also assessed using the question, “How many times in your entire life have you used these products [product name]?” with product descriptions as listed above and forced answer choices (1–2 times, 3–10 times, 11–19 times, 20–30 times, 31–99 times, 100+ times) [1,21]. Since participants could indicate use of multiple products, combustible and e-cigarette use categories were not mutually exclusive, unless explicitly stated. E-cigarette-only use was defined as having ever used any type of e-cigarette, but never used a combustible cigarette.

Loss of autonomy to tobacco products was assessed through a modified Hooked on Nicotine Checklist. HONC prompts have been modified to apply to other products [16,21,22,23] with most versions including between 9 to 11 “yes/no” items [24]. In our study, nine HONC questions were administered for each tobacco product (listed above) that the participant ever endorsed using, resulting in separate HONC scores specific to each product (Supplementary Table S1). Per standard HONC scoring, a response of “yes” to any item was coded as 1; all other responses were coded as 0. This study utilized both a binary score, indicating a loss of autonomy to nicotine, and a cumulative score, reflecting the degree of nicotine dependence [25].

### 2.3. Statistical Analysis

Descriptive statistics were used to summarize continuous and binary HONC scores by past-30-day e-cigarette and/or cigarette usage (referred to collectively as tobacco product usage). HONC scores were also examined in two subgroups: (1) e-cigarette only past-30-day users, defined as those who used an e-cigarette in the past 30 days, but had never used a combustible cigarette in their life; and (2) those who used all three e-cigarette types (disposable, pod-based, and mods/other) in the past 30 days, whether with or without combustible cigarettes. As a sensitivity analysis, HONC scores were also examined by various patterns of tobacco product usage (e.g., exclusive disposable, pod-based, or mods/other e-cigarette use), as illustrated in the flow diagram in Supplementary Figure S1. Paired *t*-tests were used to determine differences in mean HONC scores. The proportion of participants with nicotine dependence (HONC ≥ 1) by number of lifetime uses of a tobacco product was also calculated and tested for linear trend.

Multivariable logistic regression was used to determine the odds of nicotine dependence (HONC ≥ 1 vs. HONC = 0) in past-30-day disposable, pod-based, and mods/other e-cigarette users by dual-user status (e-cigarette only vs. e-cigarette/combustible cigarette dual-user) and by number of e-cigarette devices used in the past 30 days (one vs. two vs. three). All models were adjusted for age, race/ethnicity, and gender, similar to previously published analyses [18].

Missing data were minimal (<3%) across all variables used in this study, and therefore expected to have a negligible effect on our results [26]. The amount missing is reported in footnotes where relevant, and percentages are calculated with missing data in the denominator. All analyses were conducted in Stata, version 15 (StataCorp LLC, College Station, TX, USA). A *p* < 0.05 was considered statistically significant.

## 3. Results

The final sample had 4351 participants; this study focuses on 1117 participants who had used some kind of e-cigarette device in the past 30 days and 652 who had used combustible cigarettes in the past 30 days. Dual e-cigarette and combustible cigarette use was high, with 1178 participants endorsing having ever used both products. Among 1117 past-30-day e-cigarette users, 715 (64.0%) had used a disposable e-cigarette, 793 (71.0%) had used a pod-based e-cigarette, and 492 (44.0%) had used mods/other e-cigarettes. Further sub-group analyses were conducted on 381 past-30-day e-cigarette only users who had never used a combustible cigarette, and 260 participants who used all three types of e-cigarettes (disposable, pod-based, and mods/other), whether with or without combustible cigarettes, in the past 30 days. The majority of past-30-day e-cigarette users in our total sample were female (58.3%), with a mean age of 19.5 (SD 2.8); 48.6% were non-Hispanic White, 19.9% Hispanic, 15.0% African American/non-Hispanic Black, 6.8% Asian/Native Hawaiian or non-Hispanic Pacific Islander, and 9.8% identified as other/multiracial non-Hispanic. Other sample characteristics by type of e-cigarette device used can be found in Supplementary Table S2.

Endorsement of HONC symptoms among past-30-day e-cigarette users was compared to past-30-day combustible cigarette users, as seen in Table 1. Among 652 combustible cigarette users, 7.7% showed no nicotine dependence (HONC = 0), almost all (91.4%) showed signs of nicotine dependence with at least one HONC symptom, and 8.7% endorsed all 9/9 of the HONC symptoms. Patterns of nicotine dependence were similar among past-30-day e-cigarette users across all device types: for those using disposable e-cigarettes, 17.8% showed no nicotine dependence, 80.7% endorsed at least one HONC symptom, and 7.3% endorsed all 9/9 symptoms; for those using pod-based e-cigarettes, 15.1% showed no nicotine dependence, 83.1% endorsed at least one HONC symptom, and 9.3% endorsed 9/9 symptoms; for those using mods/others, 15.4% showed no signs of nicotine dependence, 82.5% endorsed at least one HONC symptom, and 5.7% endorsed 9/9 HONC symptoms. Cigarette users endorsed an average of 4.7 (SD 2.7) HONC symptoms; e-cigarette users endorsed an average of 3.9–4.1 (SDs 2.8–3.0) HONC symptoms, depending on device type.

When analyses were restricted to 381 past-30-day users who have only used e-cigarettes and never used combustible cigarettes, 72.2% of disposable users (*n* = 234), 73.6% of pod-based users (*n* = 265), and 79.0% of mods/other users (*n* = 124) endorsed at least one HONC symptom. The average number of HONC symptoms was around three for all e-cigarette devices (Table 1). Within-individual variations in HONC scores were examined in 260 participants who had used disposables, pod-based, and mods/other e-cigarettes in the past 30 days. Among these participants, the mean number of HONC symptoms for pod-based e-cigarettes (4.5, SD 2.8) was significantly higher than disposables (4.2, SD 2.8) and other/mods (4.1, SD 3.0, *p* < 0.05 and *p* < 0.01, respectively). HONC distributions by different patterns of tobacco product use (e.g., exclusive disposable, pod-based, or mods/other e-cigarette use, exclusive cigarette use) were similar to patterns presented in Table 1 and can be found in Supplementary Table S3.

“Felt like you really needed the device” was the most endorsed HONC symptom among combustible cigarette (56.9%), disposable (49.8%) and pod-based (52.2%) e-cigarette users, while “Tried to quit, but could not” was the most endorsed HONC symptom among mods/other e-cigarette users (50.0%) (Supplementary Figure S2).

The proportion of participants endorsing nicotine dependence increased with number of lifetime uses (*p*_trend_ < 0.001 for all tobacco products), with between 77.8% to 92.8% of those who used their respective tobacco product more than 100+ times in their life endorsing at least one HONC symptom (Table 2). Furthermore, increasing use of multiple e-cigarette devices was associated with increased odds of nicotine dependence in pod-based users, but this relationship was not seen in disposable or mods/other users. Among past-30-day pod-based e-cigarette users, using two types of devices was associated with a 1.71 increased odds of nicotine dependence (95% CI: 1.09–2.69), while using three types of devices was associated with a 2.38 increased odds of nicotine dependence (95% CI: 1.43–3.97). Dual-use of combustible cigarettes and e-cigarettes was also associated with increased odds of nicotine dependence among past-30-day disposable (Odds Ratio (OR): 1.92, 95% Confidence Interval (CI): 1.28–2.90) and pod-based e-cigarette users (OR: 2.53, 95% CI: 1.69–3.79). The OR among other/mod users was 1.20 (95% CI: 0.69–2.09); however, this result was not statistically significant (Table 3). Demographic variables such as age, gender, race/ethnicity, and US region were adjusted for, but were not found to contribute significantly to the model.

## 4. Discussion

This study provides comprehensive data on the presentation and extent of nicotine dependence among adolescent and young adult e-cigarette users by device type, using a large, national sample. Our results show that the majority of disposable, pod-based, and mods/other e-cigarette past-30-day users in our sample show signs of nicotine dependence, at levels comparable to cigarette users. The proportion of AYA showing at least one sign of nicotine dependence was high (80.7–83.1%) among all types of e-cigarette devices, at levels slightly higher than previously published studies using the HONC [13] and other measures of nicotine dependence [27]. Furthermore, a small but concerning percentage of AYA past 30-day users endorsed serious nicotine dependence (9/9 HONC symptoms) to e-cigarettes.

This study did not find differences in HONC dependence by e-cigarette device type, which was inconsistent with prior research where AYA who primarily used pod-based devices endorsed more HONC symptoms than those who used other e-cigarettes [18]. However, this difference may be attributed to larger sample sizes in this study and differences in study design. Further, when examining within individual differences in HONC scores among those who had used all three types of e-cigarette devices in the past 30 days, mean number of HONC symptoms was significantly higher for pod-based e-cigarettes compared to mods/other e-cigarettes. Interestingly, the mean number of HONC symptoms for pod-based e-cigarettes was also higher than disposables, which utilize similar high-concentration nicotine-salt formulas and should theoretically have the same addictive potential. It is unclear why we found greater HONC symptoms for pod-based devices over disposables. It is possible that in this particular subgroup, participants preferred pod-based e-cigarettes, with a higher proportion of participants endorsing using pod-based e-cigarettes 100+ times in their life compared to using disposables. It is also possible that these participants started using pod-based e-cigarettes earlier than disposables, and have been using pod-based devices for a longer period of time.

Causes of nicotine dependence are multifactorial, and other parameters such as frequency of use and use of multiple products may affect the likelihood of addiction, alongside device type. The proportion of participants who endorsed at least one HONC symptom increased linearly the more times a tobacco product was used in one’s lifetime. Furthermore, using multiple e-cigarette devices was associated with increased odds of nicotine dependence among past-30-day disposable and pod-based e-cigarette users. Dual use of cigarettes and any type of e-cigarette was also significantly associated with increased odds of nicotine dependence. These associations did not hold for mods/other e-cigarettes, possibly due to wider variability in nicotine delivery from adjusting settings on mod e-cigarettes, or simply from smaller sample sizes in this subgroup. Further research is needed to assess the directionality of this relationship, as it is currently unknown if increasing use of multiple e-cigarette devices in the past 30 days leads to nicotine dependence, or if nicotine dependence drives the use of multiple tobacco products to satisfy nicotine needs. Nonetheless, increasing exposure to nicotine, whether through greater number of times used, use of multiple e-cigarette devices within the past 30 days, or dual use of cigarettes and e-cigarettes, confers a greater likelihood of AYA nicotine dependence.

Of note, a large proportion of e-cigarette only users in our study also demonstrated nicotine dependence, corroborating results from previous, smaller studies [16,17]. A common argument for the safety of e-cigarettes is that adolescents who use e-cigarettes frequently usually already have had previous nicotine exposure in the form of current or former cigarette smoking [28]. Our results, however, show that signs of nicotine dependence can be found among adolescents who exclusively use e-cigarettes. A prospective study using the HONC found that e-cigarette dependence symptoms were associated with 2.56 greater odds of e-cigarette use at six months follow-up [16]. Ultimately, our results challenge some beliefs that e-cigarette usage among adolescents is mostly experimental and non-addictive. Instead, our findings show that AYA can become dependent on e-cigarettes.

E-cigarette use among AYA is a global phenomenon; while most studies of nicotine dependence have been conducted in the US, symptoms of nicotine dependence have also been observed among adolescents from Canada and the United Kingdom [29]. Further, data from US and international studies suggest that some adolescents desire to quit e-cigarettes, but have struggled to quit [29,30,31]. Given the extent of nicotine dependence among AYA, future research and resources should be dedicated towards developing and providing e-cigarette-specific cessation tools for AYA.

### Limitations

This study has several limitations. First, these findings come from a national convenience sample that recruited for e-cigarette usage and are subject to selection bias. These findings are not generalizable outside our study population. Second, this dataset was collected during COVID-19 stay-at-home orders, altering usual patterns of AYA e-cigarette usage and potentially affecting patterns of nicotine dependence. Third, while we have considered the role of exclusive use, dual cigarette/e-cigarette use, and multiple e-cigarette device use in nicotine dependence, other factors such as frequency of use (e.g., daily use, using on ≥20 of the past 30 days), customization of product, poly-substance use, or even a user’s genetic predisposition for nicotine dependence were not accounted for in this study. Lastly, nicotine dependence was measured by self-report, and HONC scores do not constitute a clinical diagnosis of nicotine dependence. However, the HONC was chosen for its flexibility in application to other tobacco products, as seen in previous studies [16,17,18,21], and has been validated biochemically [13].

## 5. Conclusions

To date, this is the largest known study on nicotine dependence among AYA e-cigarette users. In our study population, the majority of AYA who have used an e-cigarette in the past 30 days, whether with or without also using a combustible cigarette, showed signs of nicotine dependence, across all types of e-cigarette devices. A similar proportion of those who exclusively used e-cigarettes also endorsed nicotine dependence.

## Figures and Tables

**Table 1:** HONC scores among past 30-day combustible cigarette and e-cigarette users, by device type (*n* ^1^, %).

	Past 30-Day Use				E-Cigarette Only ^2^ Past 30-Day Use		
	Combustible Cigarettes (*n* = 652)	Disposable E-Cigarettes (*n* = 715)	Pod-Based E-Cigarettes (*n* = 793)	Mods/Other E-Cigarettes (*n* = 492)	Disposable E-Cigarettes (*n* = 234)	Pod-Based E-Cigarettes (*n* = 265)	Mods/Other E-Cigarettes (*n* = 124)
**HONC score**							
0	50 (7.7%)	127 (17.8%)	120 (15.1%)	76 (15.4%)	59 (25.2%)	63 (23.8%)	24 (19.4%)
1	58 (8.9%)	87 (12.2%)	82 (10.3%)	60 (12.2%)	30 (12.8%)	29 (10.9%)	18 (14.5%)
2	63 (9.7%)	54 (7.6%)	73 (9.2%)	46 (9.3%)	16 (6.8%)	27 (10.2%)	15 (12.1%)
3	54 (8.3%)	49 (6.9%)	73 (9.2%)	38 (7.7%)	14 (6.0%)	21 (7.9%)	10 (8.1%)
4	79 (12.1%)	78 (10.9%)	78 (9.8%)	52 (10.6%)	28 (12.0%)	22 (8.3%)	10 (8.1%)
5	72 (11.0%)	72 (10.1%)	78 (9.8%)	61 (12.4%)	22 (9.4%)	27 (10.2%)	14 (11.3%)
6	76 (11.7%)	59 (8.3%)	69 (8.7%)	37 (7.5%)	13 (5.6%)	19 (7.2%)	11 (8.9%)
7	80 (12.3%)	66 (9.2%)	66 (8.3%)	57 (11.6%)	18 (7.7%)	17 (6.4%)	11 (8.9%)
8	57 (8.7%)	60 (8.4%)	66 (8.3%)	27 (5.5%)	16 (6.8%)	17 (6.4%)	7 (5.6%)
9	57 (8.7%)	52 (7.3%)	74 (9.3%)	28 (5.7%)	12 (5.1%)	16 (6.0%)	2 (1.6%)
**Nicotine-dependent** **(HONC ≥ 1)**	596 (91.4%)	577 (80.7%)	659 (83.1%)	406 (82.5%)	169 (72.2%)	195 (73.6%)	98 (79.0%)
**Average (SD)**	4.7 (2.7)	3.9 (3.0)	4.1 (3.0)	3.9 (2.8)	3.4 (3.0)	3.4 (3.0)	3.3 (2.7)

Missing HONC data accounted for <2.7% of each category and were omitted from the table. ^1^ Categories are not mutually exclusive: participants who used multiple products could select all-that-apply and were counted for each device or product they use. HONC scoring was specific to each individual device or product. ^2^ Refers to participants who have only ever used e-cigarettes and have never used a combustible cigarette (*n* = 381).

**Table 2:** Participants endorsing nicotine dependence (HONC ≥1), by number of lifetime uses (*n*, %).

	Number of Lifetime Uses						
	1–2 Times	3–10 Times	11–19 Times	20–30 Times	31–99 Times	100+ Times	*p-Trend* ^1^
**Cigarettes**	270 (48.6)	201 (65.9)	85 (83.3)	71 (79.8)	53 (84.1)	181 (92.8)	<0.001
**Disposables**	180 (54.7)	178 (58.2)	93 (63.3)	101 (73.7)	73 (76.8)	188 (87.9)	<0.001
**Pod-based**	192 (43.0)	201 (53.0)	104 (62.3)	126 (76.8)	118 (76.1)	289 (89.8)	<0.001
**Mods/Others**	179 (49.7)	158 (58.1)	73 (55.3)	72 (64.9)	59 (67.0)	137 (77.8)	<0.001

Products are not mutually exclusive. ^1^ Test for linear trend of proportions. % are column percentages of participants with HONC ≥ 1.

**Table 3:** Odds ratio of HONC dependence (HONC ≥ 1) by increasing number of e-cigarette devices used in the past 30 days and dual use.

	Disposables	Pod-Based E-Cigarettes	Mods/Others
**Number of e-cigarette devices**			
1	*reference*	*reference*	*reference*
2	1.45 (0.92–2.30)	1.71 (1.09–2.69) *	2.29 (1.10–4.80) *
3	2.28 (1.37–3.79) *	2.38 (1.43–3.97) *	1.60 (0.86–2.98)
**Dual-user status**			
E-cigarette only	*reference*	*reference*	*reference*
Dual user (E-cigarette/Cigarette)	1.92 (1.28–2.90) *	2.53 (1.69–3.79) *	1.20 (0.69–2.09)

All models adjusted for age, race/ethnicity, and gender. * *p* < 0.05.

## Data Availability

Not applicable.

## Supplementary Materials

The following supporting information can be downloaded at: https://www.mdpi.com/article/10.3390/ijerph19105846/s1, Figure S1: Flow diagram by patterns of tobacco product use; Figure S2: Prevalence of Individual HONC Symptoms, by device (%); Table S1: Modified Hooked On Nicotine Checklist; Table S2: Participant Characteristics (*n*, %); Table S3: Participants endorsing nicotine dependence (HONC ≥ 1), by number of life-time uses (*n*, %).

## Abbreviations

Adolescents and Young Adults (AYA), Hooked on Nicotine Checklist (HONC), Odds Ratio (OR), Confidence Interval (CI).
