# Supplementary material for: Nicotine Dependence from Different E-Cigarette Devices and Combustible Cigarettes among US Adolescent and Young Adult Users
Source: Int J Environ Res Public Health. 2022 May 11;19(10):5846. doi: 10.3390/ijerph19105846 (PMC9140375; doi:10.3390/ijerph19105846)

Supplementary Tables and Figures

Supplementary Table S1. Modified Hooked On Nicotine Checklist

Question	Product List
Have you ever **TRIED TO QUIT** the product(s) below but couldn't?	Cigarette, even 1 or 2 puffs<br>Disposable pod-based vape like Puffbar or FOGG, even 1 or 2 puffs<br>Pod-based vape like Juul or Phix, even 1 or 2 puffs<br>Any other vape like mods, even 1 or 2 puffs
Do you use any of the products below **now** because it is really hard to quit?	Cigarette, even 1 or 2 puffs<br>Disposable pod-based vape like Puffbar or FOGG, even 1 or 2 puffs<br>Pod-based vape like Juul or Phix, even 1 or 2 puffs<br>Any other vape like mods, even 1 or 2 puffs
Have you ever **FELT LIKE YOU WERE ADDICTED** to using the products below?	Cigarette, even 1 or 2 puffs<br>Disposable pod-based vape like Puffbar or FOGG, even 1 or 2 puffs<br>Pod-based vape like Juul or Phix, even 1 or 2 puffs<br>Any other vape like mods, even 1 or 2 puffs
Have you ever felt like you **REALLY NEEDED** the products below?	Cigarette, even 1 or 2 puffs<br>Disposable pod-based vape like Puffbar or FOGG, even 1 or 2 puffs<br>Pod-based vape like Juul or Phix, even 1 or 2 puffs<br>Any other vape like mods, even 1 or 2 puffs
Do you ever have **STRONG CRAVINGS** for the products below?	Cigarette, even 1 or 2 puffs<br>Disposable pod-based vape like Puffbar or FOGG, even 1 or 2 puffs<br>Pod-based vape like Juul or Phix, even 1 or 2 puffs<br>Any other vape like mods, even 1 or 2 puffs
Is it hard to keep from using any of the products below where you are not supposed to (like in school or at home)?	Cigarette, even 1 or 2 puffs<br>Disposable pod-based vape like Puffbar or FOGG, even 1 or 2 puffs<br>Pod-based vape like Juul or Phix, even 1 or 2 puffs<br>Any other vape like mods, even 1 or 2 puffs
If you have tried to stop using product(s) below or if you have not used these product(s) for a while…Did you find it hard to concentrate because you could not use the product(s)?	Cigarette, even 1 or 2 puffs<br>Disposable pod-based vape like Puffbar or FOGG, even 1 or 2 puffs<br>Pod-based vape like Juul or Phix, even 1 or 2 puffs<br>Any other vape like mods, even 1 or 2 puffs
If you have tried to stop using product(s) below or if you have not used these product(s) for a while…Did you feel more irritable because you could not use the product(s)?	Cigarette, even 1 or 2 puffs<br>Disposable pod-based vape like Puffbar or FOGG, even 1 or 2 puffs<br>Pod-based vape like Juul or Phix, even 1 or 2 puffs<br>Any other vape like mods, even 1 or 2 puffs
If you have tried to stop using product(s) below or if you have not used these product(s) for a while…Did you feel nervous, restless, or anxious because you couldn't use the product(s)?	Cigarette, even 1 or 2 puffs<br>Disposable pod-based vape like Puffbar or FOGG, even 1 or 2 puffs<br>Pod-based vape like Juul or Phix, even 1 or 2 puffs<br>Any other vape like mods, even 1 or 2 puffs

Supplementary Table S2. Participant Characteristics (*n*, %)

	Total Sample	Past 30-day E-cigarette Users			
		All E-cigarettes[1]	Disposables	Pod-based	Mods/Others
n	4351	1117	715	793	492
**Age (mean, SD)**	19.1 (2.9)	19.5 (2.8)	19.5 (2.8)	19.5 (2.7)	20.0 (2.8)
**Gender**					
Female	2832 (65.1)	651 (58.3)	424 (59.3)	447 (56.4)	261 (53.0)
Male	1421 (32.7)	441 (39.5)	276 (38.6)	328 (41.4)	220 (44.7)
Other	97 (2.2)	24 (2.1)	14 (2.0)	17 (2.1)	10 (2.0)
**Race/Ethnicity**					
White, non-Hispanic	2611 (60.0)	543 (48.6)	356 (49.8)	379 (47.8)	214 (43.5)
Hispanic, non-AA/black	663 (15.2)	222 (19.9)	143 (20.0)	161 (20.3)	112 (22.8)
AA/black non-Hispanic	602 (13.8)	167 (15.0)	100 (14.0)	121 (15.3)	89 (18.1)
Asian/Native Hawaiian or Pacific Islander, non-Hispanic	210 (4.8)	76 (6.8)	44 (6.2)	57 (7.2)	28 (5.7)
Other/multiracial, non-Hispanic	265 (6.1)	109 (9.8)	72 (10.1)	75 (9.5)	49 (10.0)
**Region**					
South	1505 (34.6)	411 (36.8)	262 (36.6)	298 (37.6)	192 (39.0)
Northeast	909 (20.9)	252 (22.6)	164 (22.9)	184 (23.2)	109 (22.2)
West	990 (22.8)	244 (21.8)	158 (22.1)	165 (20.8)	106 (21.5)
Midwest	918 (21.1)	197 (17.6)	124 (17.3)	139 (17.5)	77 (15.7)
US Territories	11 (0.3)	6 (0.5)	2 (0.3)	5 (0.6)	2 (0.4)

[1]Participants who indicated past 30-day use of disposable, pod-based, or mods/other e-cigarettes

E-cigarette categories are not mutually exclusive: participants who used multiple products could select all-that-apply and were counted for each device or product they use.

Missing data accounted for <1% of the total sample. Percentages were calculated with missing included in the denominator

Supplementary Table S3. HONC Scores by different patterns of tobacco product use (*n*, %)										
	**Never used an E-cigarette**	**Never used a Cigarette, Past 30-day use of only one e-cigarette device**			**Never used a Cigarette or >1 e-cigarette device, exclusive past 30-day use of one e-cigarette device**			**Past 30-day use of disposables, pod-based and mods/other e-cigarettes**		
**HONC**	Past 30-day Cigarette Use (*n* = 64)	Disposable (*n* = 58)	Pod-based (*n* = 99)	Mods/ Other (*n* = 24)	Disposable (*n* = 22)	Pod-based (*n* = 58)	Mods/ Other (*n* = 13)	Disposable (*n* = 260)	Pod-based (*n* = 260)	Mods/ Other (*n* = 260)
**0**	6 (9.4)	21 (36.2)	34 (34.3)	5 (20.8)	5 (22.7)	21 (36.2)	3 (23.1)	32 (12.3)	27 (10.4)	41 (15.8)
**1**	2 (3.1)	10 (17.2)	8 (8.1)	3 (12.5)	5 (22.7)	3 (5.2)	1 (7.7)	31 (11.9)	26 (10.0)	29 (11.2)
**2**	8 (12.5)	3 (5.2)	13 (13.1)	3 (12.5)	2 (9.1)	11 (19.0)	1 (7.7)	21 (8.1)	18 (6.9)	24 (9.2)
**3**	5 (7.8)	4 (6.9)	5 (5.1)	5 (20.8)	3 (13.6)	0	3 (23.1)	14 (5.4)	24 (9.2)	16 (6.2)
**4**	13 (20.3)	5 (8.6)	8 (8.1)	1 (4.2)	2 (9.1)	5 (8.6)	1 (7.7)	33 (12.7)	30 (11.5)	24 (9.2)
**5**	8 (12.5)	1 (1.7)	4 (4)	3 (12.5)	1 (4.5)	4 (6.9)	2 (15.4)	34 (13.1)	32 (12.3)	33 (12.7)
**6**	8 (12.5)	1 (1.7)	8 (8.1)	3 (12.5)	2 (9.1)	4 (6.9)	1 (7.7)	25 (9.6)	25 (9.6)	15 (5.8)
**7**	8 (12.5)	3 (5.2)	6 (6.1)	1 (4.2)	2 (9.1)	5 (8.6)	1 (7.7)	22 (8.5)	25 (9.6)	37 (14.2)
**8**	4 (6.3)	4 (6.9)	5 (5.1)	0 (0)	0 (0)	2 (3.4)	0 (0)	31 (11.9)	25 (9.6)	17 (6.5)
**9**	1 (1.6)	6 (10.3)	6 (6.1)	0 (0)	0 (0)	2 (3.4)	0 (0)	13 (5.0)	24 (9.2)	20 (7.7)
**HONC ≥1**	57 (89.1)	37 (63.8)	67 (63.6)	19 (79.2)	17 (77.3)	36 (62.1)	10 (76.9)	224 (86.2)	229 (88.1)	215 (82.7)
**Mean HONC score (SD)**	4.3 (2.4)	2.9 (3.3)	2.9 (3.0)	2.8 (2.2)	2.6 (2.4)	2.8 (2.9)	3.0 (2.3)	4.2 (2.8)	4.5 (2.8)	4.1 (3.0)
Missing data accounted for 0.0-2.0% of the data and was omitted from the table. Percentages were calculated with missing included in the denominator										

Supplementary Figure S1. Flow diagram by patterns of tobacco product use

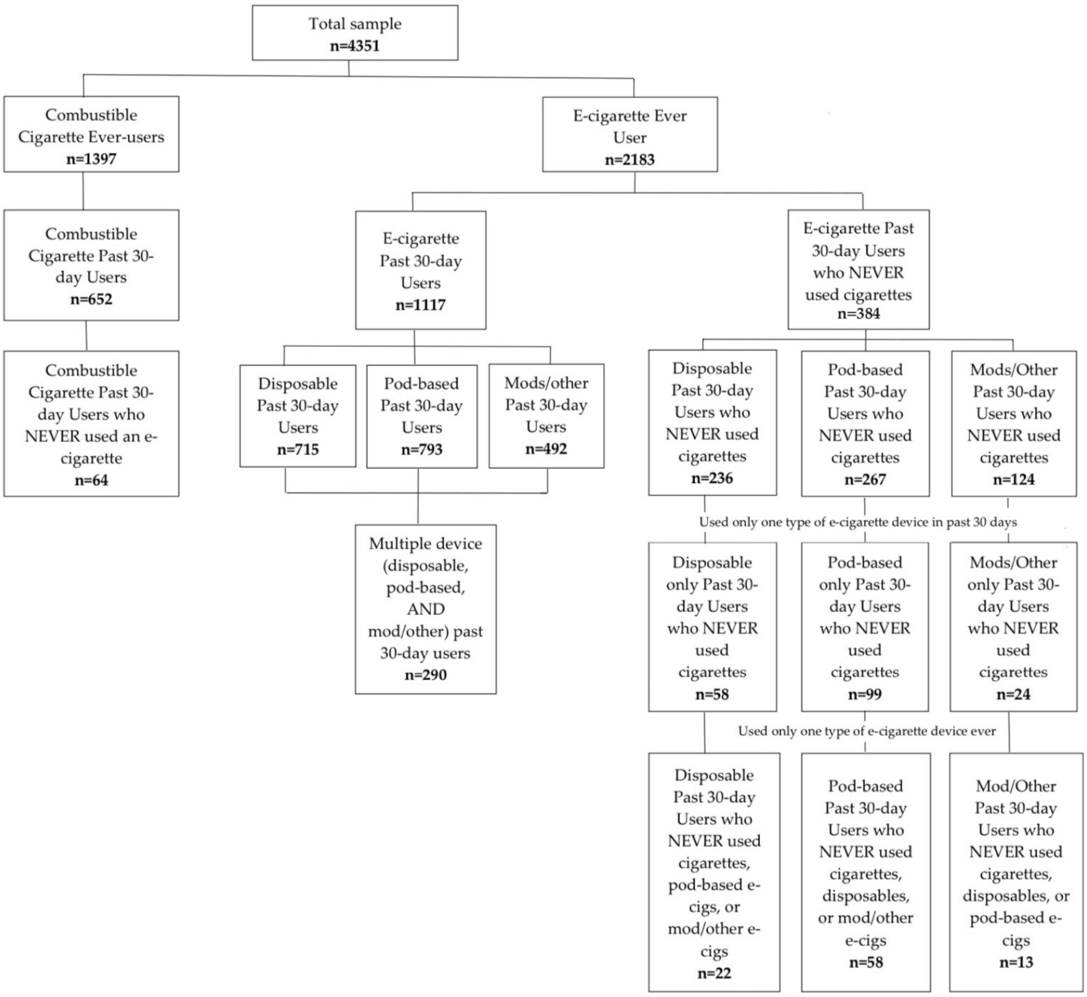

Supplementary Figure S2. Prevalence of Individual HONC Symptoms, by device

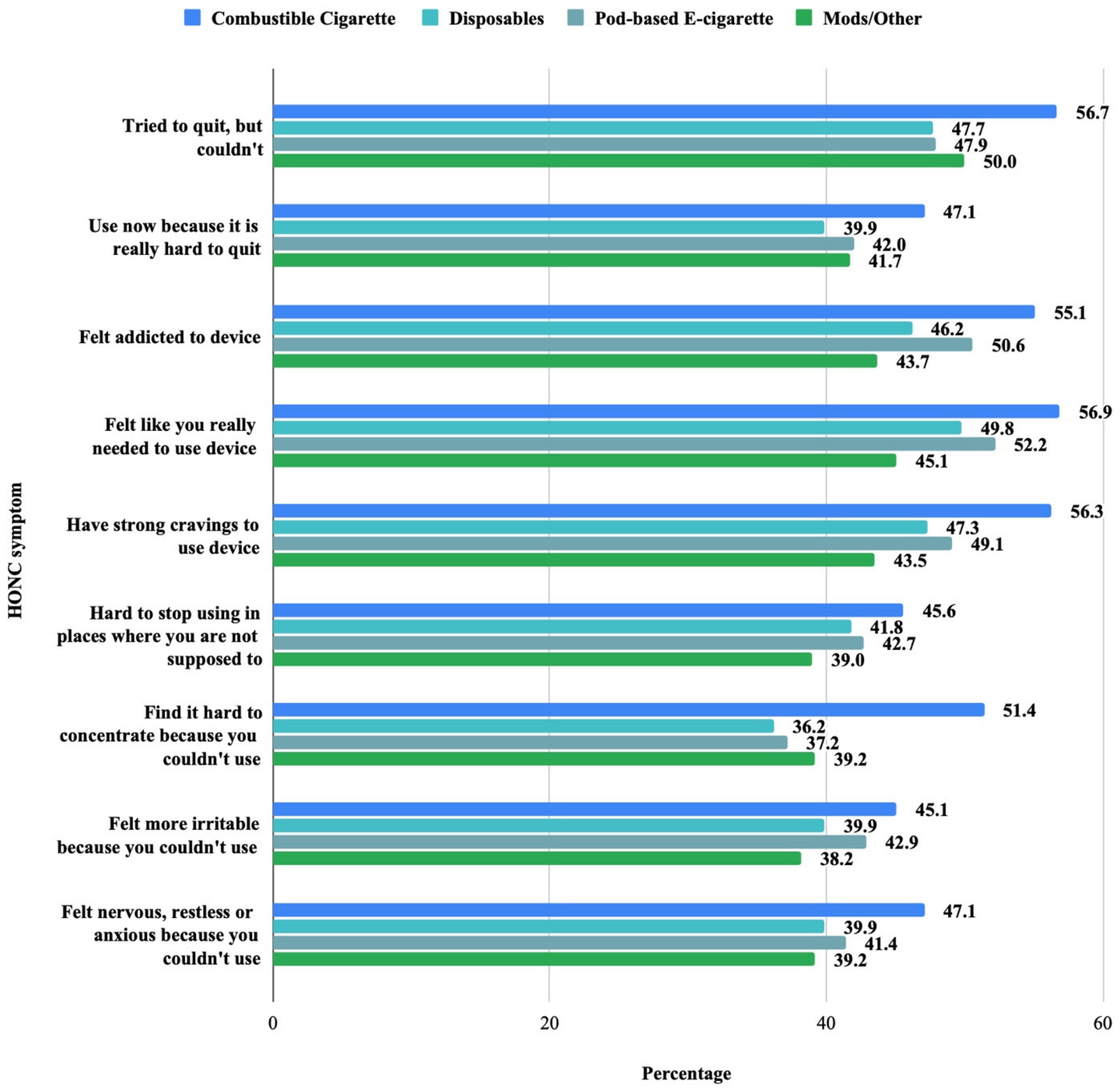

Supplement: Supplementary file 1 [file ijerph-19-05846-s001.zip › ijerph-1675332-supplementary.pdf]
